# A Simple Explanation for Harmonic Word Order

**DOI:** 10.1111/cogs.70056

**Published:** 2025-04-08

**Authors:** John Mansfield, Lothar Sebastian Krapp

**Affiliations:** ^1^ Institute for the Interdisciplinary Study of Language Evolution University of Zurich

**Keywords:** Word order, Syntax, Harmonic, Linguistic typology, Language evolution, Computational simulation, Similarity

## Abstract

Harmonic word order is a well‐established tendency in natural languages, which has previously been explained as a single ordering rule for all head‐dependent relations. We propose that it can be more parsimoniously explained as an outcome of word‐class frequencies, where the purported “head” is the most frequently instantiated word class in a phrasal schema. We show that the most frequent class gravitates spontaneously to an edge position in a phrasal replication process, as long as words of one class may influence the position of words of another class. This avoids the need to posit head‐dependent ordering as an innate rule or bias, simplifying our theory of word order. We demonstrate the spontaneous emergence of harmony from word‐class frequencies using a simple computational model of phrasal replication, and in further extensions show that the principle remains robust with fuzzy word classes and multiword chunks, can capture competition between harmony and locality, and is compatible with the results of behavioral experiments on harmonic ordering. Our findings support further exploration of syntactic models with nondiscrete word classes.

## 1 Introduction

Harmonic ordering of heads and dependents is one of the best‐known cross‐linguistic patterns in syntax (Biberauer & Sheehan, 2013). For example, a noun and its dependent modifiers tend to be arranged with all dependents on the same side, either preceding the head as in the English noun phrase (NP) *those two long fish‐nets*, or following the head as in Mauwake *mera‐sia maala erup nain* (fish‐net long two those) (Berghäll, 2016: 231). This is a case of “parallel” harmony within a single phrasal level, while other structures exhibit “hierarchical” harmony across nested levels (Jing, Blasi, & Bickel, 2022). As we will see below, it is parallel harmony that poses the more difficult challenge to our theories of word order.

In this article, we propose a simple explanation for parallel harmonic order, which has hitherto been overlooked. We show that heads may be preferentially positioned at a phrase edge simply because they are the most frequent word‐class in the phrase. We demonstrate this using a simple word ordering algorithm that samples previous phrases and replicates their structure. Crucially, this algorithm allows for certain well‐defined mismatches between a new phrase and a previous phrase. This makes the process powerful enough to produce novel orders, but also turns out to be sufficient to produce harmonic ordering. Our “replication‐with‐modification” approach is compatible with dynamic approaches to syntax, including evolutionary processes where syntax develops incrementally (Diessel, 2019; Progovac, 2015). Besides revealing a fundamental relationship between word‐class frequency and harmonic order, our model also elegantly captures the competitive relationship between harmony and locality (Gildea & Temperley, 2010).

We assume that words belong to word classes, and that the “head” can be defined as the word class that is most frequently instantiated in a phrase type. As we will see below, this is consistent with most views in the literature on heads, and has the advantage of replacing theory‐internal concepts of headedness with a clearly measurable criterion. Our most crucial assumption is that words of different classes can influence one another, for example, a previous phrase of the form X‐Y may favor a new phrase of the form X‐Z, rather than Z‐X. Words of different classes, “Y” and “Z,” preferably go in the same position relative to a constant class “X.” Similar assumptions may be required for any model of word‐order harmony, since harmony by definition involves similar positioning of distinct classes. But we will argue that this type of different‐class matching is *all* we need to explain harmony, while head‐dependent ordering rules can be jettisoned, since differential word‐class frequencies already produce the type of asymmetric phrase structures we seek to explain. Parallel harmonic order can, therefore, be restated as: *the word class with the highest frequency is at the edge of the phrase*.

The reinterpretation of headedness in terms of frequency is in line with the idea that the head of a phrase is an obligatory element (e.g., Hengeveld, Rijkhoff, & Siewierska, 2004: 530), following an older idea that the phrase as a whole has a similar syntactic distribution to the head (Bloomfield, 1933; Wells, 1947). If the head is obligatory, this implies that it should be more frequent than nonobligatory elements. In fact, heads are not strictly obligatory, as seen in examples like *the poor*, where the NP lacks a noun. But our frequency approach avoids this problem, since “the most frequent element” covers both cases of actual obligatoriness and near‐obligatoriness. A more serious limitation of headedness is the lack of theoretical clarity about what it means for a word to be the head of a phrase, or how to identify heads (Croft, 1996; Fraser, Corbett, & McGlashan, 1993). Some models of grammar treat headedness as a theoretical primitive, without explanation (e.g., Kahane & Osborne, 2015; Tesnière, 2015 [1966]: 5: lxi). Headedness may also have an important theory‐internal function (e.g., Kornai & Pullum, 1990), but this may not clarify its relevance outside of that theoretical framework. Rigorous analyses of purported head properties have found that they do not align on the same phrasal elements, which leads to fragmentation of the concept, or a range of quite different concepts sharing the same terminological label (Freywald & Simon, 2022; Lander, 2022; Zwicky, 1985). This makes the concept of headedness dangerously flexible. Thus, one advantage of our approach is that it replaces a protean concept with a clearly measurable concept, namely, frequency. We also thus avoid unresolved (or unresolvable) debates about whether head of the NP is really the noun or the determiner (Salzmann, 2020). What we are really interested in is the most frequent word class.

Since we assume the potential for interaction between different word classes, our approach is incompatible with theories of grammar in which word classes are functionally discrete categories. In much of the psycholinguistic and computational modeling literature, syntactic categories are treated as sets of discrete symbols such as {N, V, Adj…} or {S, V, O…}, but it is arguably more reasonable to allow for some type of interaction or relationship between categories. There is a substantial tradition in typological linguistics of treating word classes as gradient or “fuzzy” categories (for an overview, see Keizer, 2023), based, for example, on degrees of lexical overlap between categories, or distributional similarities between categories. In some work, individual words are treated as more or less prototypical members of syntactic categories (e.g., Auwera & Gast, 2010; Crystal, 1967), which may also imply that words of different classes can have degrees of similarity to one another. Other approaches assume that word classes are built from discrete categorical features, but each class is a composite of such features, with feature‐sharing between classes (for an overview, see Zeijlstra, 2023). While sanitized data tends to allocate each word to a unique category, in practice, linguists are not always able to decisively assign words to classes, since individual words may have characteristics of more than one class (e.g., Lyons, 1999: 34; Hurford, 2012: 309; Taylor, 2014: 183). Thus, while word classes continue to be treated as fully discrete in much of the literature, this appears to be a practical simplification rather than a motivated decision. The current study contributes to an alternative approach in which word classes are not fully discrete, in ways to be elaborated below.

Below, we will first show that, despite an extensive literature on harmonic word order, parallel harmonic order still demands a stronger theoretical explanation (Section 2). We describe the genesis of our new approach and motivate our use of a maximally simple replication process, as opposed to a more complex psycholinguistic model (Section 3). We then explain the replication‐with‐modification algorithm conceptually (Section 4), before illustrating both the outputs of a computational implementation, and some analytical mathematical results (Section 5). In the second half of the article, we explore several extensions to the basic algorithm, demonstrating its applicability beyond the NP, and its compatibility with psycholinguistic theories and phenomena (Section 6). Code, data, Supplementary Analyses, and formal mathematical description are available in an open data repository.^1^

## 2 Types of harmony, and competition with locality

**Fig. 1:**
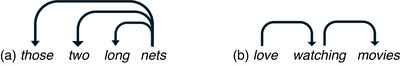
(a) Parallel harmonic order; (b) hierarchical harmonic order.

There are two distinct types of harmonic word order, parallel and hierarchical (Jing et al., 2022). Noun‐phrase examples like *those two long nets* exhibit parallel harmony (Fig. 1a), where multiple dependents of the same head are arranged in the same direction. hierarchical harmony (Fig. 1b) instead involves the dependent in one relation being the head in another relation, for example, a matrix verb with a dependent verb, which in turn has a dependent noun object.

There is substantial evidence for word‐order harmony in natural languages, though this is a statistical tendency rather than a hard constraint. Evidence has been found in typological research (Dryer, 1992; Dryer, 2018; Greenberg, 1963), and more recently, phylogenetic modeling of word‐order changes, which shows that word orders are more likely to change into harmonic configurations than disharmonic (Jäger & Wahle, 2021). The evidence for a harmonic ordering bias is found both in hierarchical structure (clause‐level dependencies tend to have the same direction as dependencies within clausal arguments), and in parallel structure (multiple noun modifiers tend to be on the same side of the noun, and multiple arguments tend to be on the same side of the verb). Arguably, the clearest parallel tendency is among the noun modifiers: adjective, number, and demonstrative words tend to be positioned on the same side of the noun in an NP (Dryer, 2018; Jäger & Wahle, 2021). There is also psycholinguistic evidence for a learning bias favoring harmonic noun‐phrases (Culbertson & Newport, 2015; Culbertson & Newport, 2017; Culbertson, Franck, Braquet, Barrera Navarro, & Arnon, 2020; Culbertson, Smolensky, & Legendre, 2012). We will see below that across diverse languages, the noun is consistently the most frequent word class in the NP, which motivates the reinterpretation of noun‐phrase harmony in terms of word‐class frequency. Throughout the article, we will take the NP as our main example, though in fact our algorithm is quite general and can be applied to other grammatical structures, including clausal ordering (Section 6.1).

Harmony interacts with another major ordering property, locality. In locality theory, language comprehension requires integrating words that are syntactically composed with one another, and this is easier when the words are in linear proximity, or “local” to one another (Gibson, 2000; Hawkins, 1994). Harmony interacts with locality in complex ways, once the aggregate dependency lengths of a sentence are taken into account (Futrell, Levy, & Gibson, 2020; Jing et al., 2022). But in simplest terms, hierarchical harmony satisfies locality, while parallel harmony violates it (Gildea & Temperley, 2010). The prolocality effect of hierarchical harmony can be seen in Fig. 1b, where each pair of words linked by a dependency is maximally local to one another. Computational modeling also supports the idea that hierarchical harmony is driven by the need to shorten dependency lengths (Christiansen & Devlin, 1997). Thus, the drive for locality may provide an explanation for hierarchical harmonic order. However, this explanation does not extend to parallel harmonic order. For example, in Fig. 1a, the harmonic ordering of parallel dependents creates an antilocality effect, with longer dependencies (Hahn & Xu, 2022). Therefore, it is specifically *parallel* harmonic order that is in stark need of theoretical explanation, and which is the focus of the current study.

Previous explanations of harmonic word order make their own claims for simplicity. One approach evokes a highly general grammatical rule, which specifies the direction of dependency linearization for all word combinations (Venneman, 1973; Venneman, 1974). A similar idea can be found in “principles and parameters” theory, where a single head‐direction parameter could theoretically reduce the complexity of grammar (Chomsky, 1981; Travis, 1984). A single direction rule could simplify grammars by avoiding the need for specific linearization rules for specific word classes, instead having just one rule covering all head‐dependent relations. However, one problem with this approach is the flexibility of the concept of “headedness,” as described above. This problem has already been acknowledged in previous work, noting, for example, that different annotation decisions about heads have the potential to drastically alter research results (Song, 2018: 245; de Marneffe & Nivre, 2019). Another problem with a generalized linearization rule is that it takes an “all or nothing” approach, where all dependencies, both parallel and hierarchical, should go in the same direction (Hawkins, 1980). But the evidence suggests that languages tend to have only a *relatively* consistent dependency linearization, rather than being wholly consistent (Dryer, 2018; Jing et al., 2022). Thus, a “total harmony” rule does not fit the data well, and we should instead seek a dynamic model that can generate probabilistic harmony.^2^

## 3 The virtue of simplicity

In this study, we offer a novel explanation for parallel harmonic order, based on the differential frequency of word classes. We show that in a simple phrasal replication algorithm, the most frequent word class naturally gravitates to an edge, offering a parsimonious explanation for parallel harmony in natural languages. But before we demonstrate this principle, it is worth clarifying some differences between our approach and other computational modeling work, and the relevance of abstract algorithms to natural language phenomena. Since our proposal has no clear precedent in syntactic theory, we also describe how we arrived at the idea that harmony is driven by word‐class frequency.

Much other computational work simulates psycholinguistic processes of syntactic learning and production (e.g., Chang, Dell, & Bock, 2006; Everbroeck, 2003; Lupyan & Christiansen, 2002; McCauley & Christiansen, 2019), or agent‐based processes of cultural transmission (e.g., Baxter, Blythe, Croft, & McKane, 2006; Blythe & Croft, 2021; Griffiths & Kalish, 2007; Motamedi, Wolters, Naegeli, Kirby, & Schouwstra, 2022; Smith & Wonnacott, 2010; Smith et al., 2017). The current study is very different to these, presenting a simple replication algorithm rather than a psycholinguistic model. Furthermore, while many computational models aim to recapitulate the exact word orders found in natural corpus data (Chang, Lieven, & Tomasello, 2008), we instead focus purely on the degree of harmony in our model outputs.

Our current approach grew out of exploratory work aiming to simulate the emergence of syntactic categories and phrase structure, while approximating psychological processes. The simulations required relatively complex models incorporating semantic similarity, memory decay, entrenchment, and chunking, and while they produced some language‐like outputs, we ultimately concluded that model complexity made the results difficult to interpret.^3^ However, we also observed that harmonic ordering emerged in almost all simulations, likely due to fuzzy matching between word classes. This led to the following hypothesis: that whenever words of different syntactic categories have an influence on each other's positioning, this predominantly affects the less‐frequent categories, leaving the most frequent category positioned at one edge of the phrase. The current study investigates this hypothesis, and in contrast to our exploratory work, aims to formalize it in the simplest possible way.

A simpler algorithm for word order more clearly demonstrates the relationship between harmony and word‐class frequency. We here present a model using the fewest possible ingredients, where phrasal ordering assumes nothing more than the consistent linear positioning of word classes (cf. Mansfield et al., 2022; Mansfield, Stoll, & Bickel, 2020), as well as the potential for interaction between classes. More complex models may better approximate natural language processes, but with more complex models, such as artificial neural networks (ANNs), it is not always completely clear *why* they work (Zhang, Tiňo, Leonardis, & Tang, 2021). Our aim in this study is to provide a stronger theoretical explanation for harmony, rather than modeling its instantiation in natural languages.

While our basic algorithm is very simple, we nonetheless claim that it is compatible with psycholinguistic processes. To support this, in the second half of the article, we demonstrate some extensions to our model that integrate fuzzy word classes and multiword chunking. In the Supplementary Analyses, we also demonstrate an extension with iterative learning. We show that our algorithm still produces harmonic order when combined with these other mechanisms. The success of these extended simulations suggests that frequency‐based harmony is indeed relevant to real‐world linguistic processes, rather than being of purely theoretical interest. We also discuss potential compatibility with psychological experiments on harmonic ordering, and with general models of syntactic learning and production. Since our notion of phrasal replication makes very few assumptions, we expect that it could be integrated into a wide range of learning and production mechanisms—essentially, any dynamic system where syntactic production replicates input data, and syntactic categories are not fully discrete.

## 4 Ordering phrases by replication‐with‐modification

In this section, we explain our model of word order on a conceptual level, laying out the theoretical primitives. We here provide a relatively informal description, while a mathematically formalized version is included in the data repository.

Let us assume that phrase structure imposes a linear order on linguistic expressions, each of which consists of one or more words belonging to a finite set of word classes, *c_1_*, *c_2_* … *c_n_*. Whichever of these classes occurs most frequently in a phrase type, we will call *c_F_*. Note that if one word class is obligatorily present in every phrase, this is just a special case of *c_F_*.^4^ Phrase structure is the consistent relative ordering of word classes in a phrase type, and parallel harmony is satisfied whenever *c_F_* is either first or last in this order. Thus, harmonic structures include [***c_F_***‐*c_1_*‐*c_2_*], [***c_F_***‐*c_2_*‐*c_1_*], [*c_1_*‐*c_2_*‐***c_F_***], and so on, while disharmonic structures include [*c_1_*‐***c_F_***‐*c_2_*], [*c_2_*‐***c_F_***‐*c_1_*], and so on. Natural language also involves hierarchical structures, where phrases are linearized inside phrases, but in this study, we focus on the problem of linearizing a simple phrase.

To explain harmonic order, rather than merely stipulating it, we develop a model where word order has no inherent fixed rules, but instead *develops* consistent patterns under the influence of other principles. Starting from a completely unstructured state, where all possible word orders are equiprobable, consistent ordering can develop from a sampling regime. Finite samples tend to under‐represent actual diversity (e.g., Chao & Shen, 2003; Meinhardt, Malouf, & Ackerman, 2022), and when we recursively take finite samples, then add them to the pool from which further samples are taken, such processes gradually converge on fewer variants. In terms of word order, sampling regimes can, therefore, converge on relatively fixed word orders. Our core process involves the replication of samples: each new expression is linearized by replicating previous phrases, or more concretely, positioning words of the same class in the same linear positions, as in *these black cats* and *those brown dogs* (Herce, Saldana, Mansfield, & Bickel, 2023; Mansfield et al., 2022). But there are limitations to any system that can only replicate based on complete one‐to‐one matching of word classes. A replication process is underpowered if it cannot order new word‐class combinations, for example, ordering an expression {N, Adj, Det}, when only expressions combining {N, Adj} and {N, Det} have been previously linearized. This requires a replication algorithm that can match a three‐word phrase against two‐word phrases. Furthermore, it may be that any model producing harmonic order *must* permit some form of matching between distinct classes, since between‐class relations are at the core of harmony. Both ordering novel expression types, and interaction between classes, can be achieved using replication‐with‐modification, where new phrases approximate sampled phrases, rather than being limited to exact matches. We need a replication algorithm that preferentially matches words of the same class, but can also match words of distinct classes. This also reflects the idea that word classes are not fully discrete, but rather have overlap and similarity between classes. In our simplest implementation, we model word classes as discrete symbols, but allow different symbols to be matched so that they are not *functionally discrete* in the replication mechanism. In an extension to the basic model, we will implement a version with fuzzy word classes (Section 6.2), where each class is a cluster in a multidimensional space, and matching is based on proximity.

**Fig. 2:**
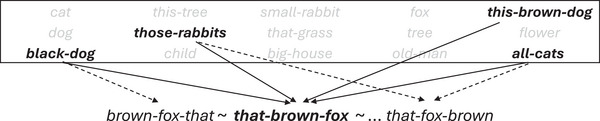
Schematic example of linearization algorithm. The box contains previous phrases, of which those in bold are being sampled for the replication process. Variant linearizations of the target phrase are shown below, with the selected variant in bold. Matches with the selected variant are indicated by solid lines; matches with other variants by dashed lines. Here, the variant *that‐brown‐fox* is selected, as it matches the greatest number of sampled phrases.

Now, let us see why replication‐with‐modification gives rise to word orders with the most frequent word class at one edge. An unordered expression like {*brown*, *fox*, *that*} is linearized by selecting one possible order, either *brown‐fox‐that*, *that‐brown‐fox* … and so on. Many such expressions are linearized, one after another, and the linearization of each new expression is influenced by a sample of previous phrases, as schematized in Fig. 2. The new expression {*brown*, *fox*, *that*} is influenced by the sampled phrases, such that the linear order of each sample will influence the linearization of the new expression. Once the new expression has been compared to the samples, whichever variant matches the most samples is selected as the linear output for the new phrase. In our example, the variant *that‐brown‐fox* is selected, as it matches the most samples. The alternative linearizations *brown‐fox‐that* and *that‐fox‐brown* also match some samples. Matching requires compatible word orders, to be defined below. In this example, the unsuccessful variants matched some two‐word phrases, but the successful variant received more matches, since it matched the same two‐word phrases, and additionally the three‐word phrase *this‐brown‐dog*.

What does it mean for an ordering variant to match a sampled phrase? Each word in the new expression can match with at most one word in a sampled phrase, and this word is preferentially of the same word class, though different‐class matching is also possible. In the demonstrations below, we will implement this in two different ways. First, we will use the simplest possible model, where word‐classes are represented as discrete labels {N, Adj, Num, Det}, but they are not functionally discrete, because the matching algorithm allows different‐class matches once all possible same‐class matches have been exhausted. Second, we will demonstrate a version where word classes are clusters of points in a multidimensional space, such that words of the same class tend to be closer to one another. Matching by proximity then results in preferential same‐class matching, while also allowing for occasional different‐class matching.

Matched words establish compatibility of linearization between a sampled phrase and an ordering variant. Compatibility is satisfied if, for each pair of words in the sampled phrase {X_S_, Y_S_}, which are matched with a pair of words in the new expression {X_T_, Y_T_}, the sampled linear precedence relation X_S_>Y_S_ is the same as the variant precedence relation, X_T_>Y_T_. Note that this does not require adjacency between words, but only linear precedence. The effect of this is that a Det‐Adj‐N sample phrase would, for example, match a variant Det‐N, given that the matching {Det, N} pairs have the same precedence relation, Det>N. This follows standard grammatical analyses, where an expression like *that‐fox* is treated as compatible with schemas such as Det‐Adj‐N.

If words preferentially match to their own class, but can also match to a different class, then all else being equal, a class that occurs more frequently will have more same‐class matches. Less frequent classes tend to have more different‐class matches, since they have a lower probability of finding same‐class matches in a sampled phrase. In NPs, if the N class is consistently present, and other classes such as Adj and Det are only intermittently present, then different‐class matching will tend to occur more between Adj and Det, rather than between N and Adj, or N and Det. Classes that receive more different‐class matching will influence each other so that they tend to be positioned on the same side of a class that receives more same‐class matching. Furthermore, incomplete matches also allow for samples such as Det‐N and Adj‐N to both provide matches for a new phrase Det‐Adj‐N. Thus, differences of class frequency give rise to the characteristic edge position of so‐called “heads.”

## 5 The emergence of harmony from corpus data

In this section, we implement the phrasal replication model outlined above, demonstrating that harmonic ordering emerges spontaneously in the vast majority of phrases. The computer code for the implementation is available at the data repository.^5^ We here implement word‐order replication by taking NPs as an example, while a subsequent section demonstrates an implementation on clausal ordering (Section 6.1).

We extract the word‐class combinations from NPs in natural corpus data, and use these as material for implementing phrasal replication. For example, from a corpus phrase *three black cats*, we extract an unordered class combination, {Adj, N, Num}. We extract such combinations from the Universal Dependencies v.2.13 corpus collection (de Marneffe, Manning, Nivre, & Zeman, 2021), thus producing for each language a (randomly shuffled) series of expressions, each of which will be linearized by replication of previous phrases. Corpora yielding less than 100 NPs were excluded, while for larger corpora, we select a random sample of 1000 expressions, since this is more than sufficient for consistent ordering to emerge. This provides corpora for 107 languages from 20 language families, with Indo‐European represented by many languages, and most other families represented by a single language. For the illustrations below, we use a selection of 30 languages: one randomly selected from each of the 20 families, plus another 10 selected from Indo‐European. We include extra Indo‐European corpora because they tend to have higher NP complexity, which provides a more strenuous test for the model. Results for all languages are in the Supplementary Analyses.

### 5.1 Statistical properties of NPs in natural corpora

**Fig. 3:**
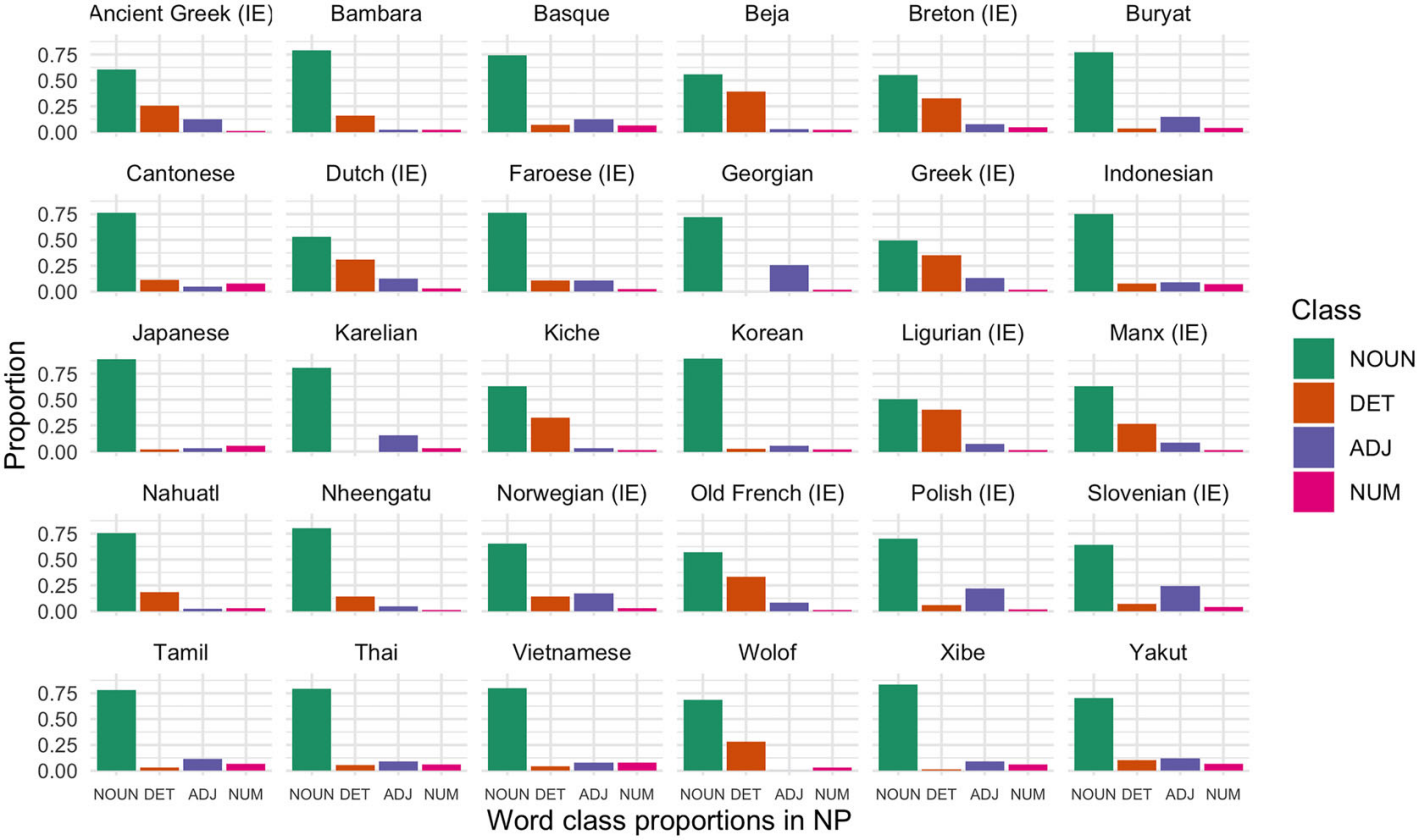
Word class proportions in NPs for the 30 illustrative corpora. In most languages, nouns are much more frequent than any other word class.

NPs in the sample corpora have a characteristic statistical profile, which plays an important role in the emergence of harmony. On the one hand, nouns are by far the most frequent word class in NPs from all corpora, supporting the reinterpretation of “headedness” in terms of word‐class frequency. This is illustrated in Fig. 3. For many languages such as Bambara and Japanese, nouns make up around 75–80% of all words in NPs. Determiners are usually the next‐most‐frequent class, although there are a few languages such as Georgian and Polish where adjectives are the next‐most‐frequent. Indo‐European languages tend to have more frequent non‐noun classes, especially determiners, which in some languages like Greek and Ligurian are almost as frequent as nouns, though never as frequent.

**Fig. 4:**
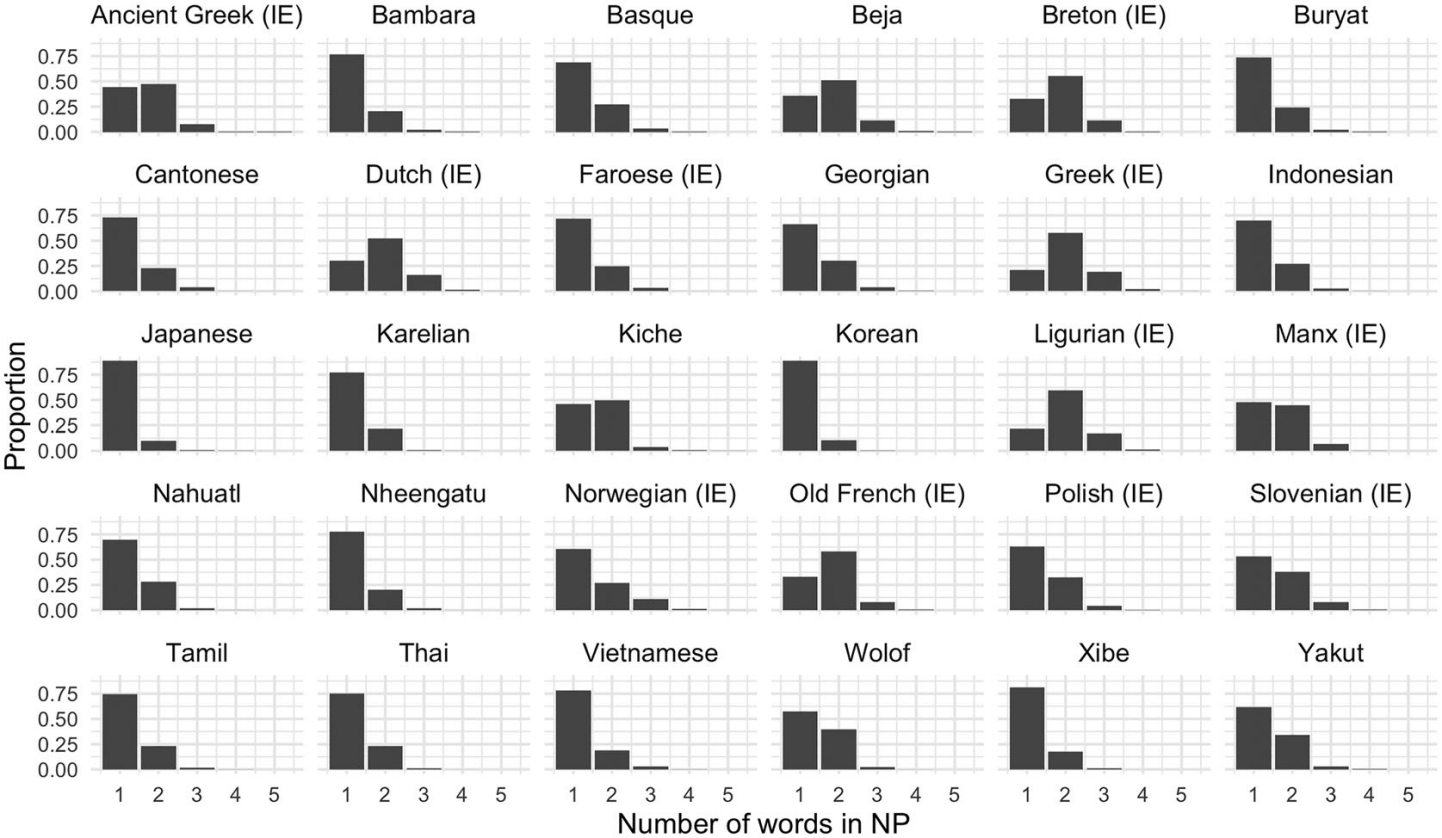
NP complexity for the 30 illustrative corpora. In all languages, typical NP complexity is either one word or two words. NPs longer than three words are very rare (note that this excludes relative clauses and genitives).

Not only are nouns the most frequent word class, often by a large margin, but even the sum of non‐noun words in each NP is on average relatively low (note that hierarchical structures with genitives and relative clauses have been excluded from consideration). The number of words per NP is illustrated in Fig. 4. For most languages, one‐word (noun‐only) NPs are the most frequent, with a sharp monotonic decrease for additional words. These languages are in line with previous findings that phrase length is approximately Zipfian (Piantadosi, 2014). There are also some languages where two‐word NPs are more frequent, and in these, the additional word is most frequently a determiner. The two‐word type is dominant in many Indo‐European languages with high‐frequency determiners (e.g., Greek, Ligurian), but it is also found in Beja (Afro‐Asiatic) and K'iche’ (Mayan).

In summary, nouns are consistently the most frequent word class, and phrases with few words are more frequent than phrases with many words. Under our replication model, the combination of these statistical properties favors noun positioning at the edge of the phrase. If most phrases were to contain two or more non‐noun words, then replication could just as well favor orders such as Det‐N‐Adj, since the classes Det and Adj would have more same‐class matching and less different‐class matching. But since multiword phrases usually have exactly two words, matching is more often between expressions like {N, Det} and {N, Adj}, which favor harmonic ordering.

### 5.2 Computational implementation and results

Taking the corpus data illustrated above as input, we implement the phrase ordering algorithm for each language in the dataset. Within each language, we take our sample of up to 1000 phrases and linearize each in turn, repeating this procedure 100 times, thus completing 100 “iterations” for each language. Within each iteration, each new phrase is linearized by taking a sample of previous phrases in the same iteration, generated by giving each previous phrase an independent 0.5 probability of inclusion in the sample. Using a probabilistic sample, rather than sampling all previous phrases, makes the model more psychologically plausible while also greatly reducing computational runtime. At the beginning of an iteration, the set of previous‐phrase samples is empty, in which case, the current phrase is output with random order. The process thus begins from a point of randomness, and gradually develops consistent ordering as the pool of samples grows larger.

Before showing the overall results, it is worth showing some snapshots from the progress of an iteration, to better understand how it works. Fig. 5a illustrates an early point in an iteration over the Norwegian corpus. Inside the box are 14 phrases that have already been linearized, of which six are now being randomly sampled, and below the box are the two potential linearization variants for a new expression {N, Det}. Of the randomly sampled previous phrases, only the multiword phrases have the potential to discriminate between variants. In this case, both multiword samples, Adj‐N and Num‐N, match the new expression by same‐class matching N:N, and different‐class matching Adj:Det and Num:Det. The ordering of both these samples is compatible only with the variant Det‐N, which is, therefore, selected.

**Fig. 5a:**
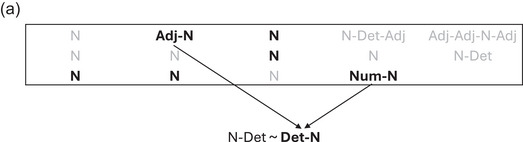
Linearization after 14 phrases of the Norwegian corpus. The box contains previous phrases, of which those in bold are being sampled for the replication process. Variant linearizations of the target phrase are shown below, with the selected variant in bold. Matches to the selected variant are indicated by solid lines. In this case, the unselected variant N‐Det did not receive any matches.

Fig. 5b shows the next multiword expression from the same iteration, which now has a slightly larger sample pool. This time the sampled phrases do not concur on a single output variant, and dashed lines indicate matches to ultimately unsuccessful variants. The same number of matches go to a harmonic variant, Adj‐Det‐N, and a disharmonic variant, Det‐N‐Adj, resulting in a randomized tie‐breaker, which in this instance selects the latter. Disharmonic outcomes such as this tend to occur more often in the earlier stages of an iteration, when the sample pool is smaller, and consistent orders have not yet emerged.

**Fig. 5b:**
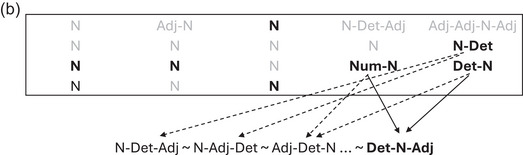
Linearization after 18 phrases of the Norwegian corpus. Here, several variants receive some matches, with matches to the selected variant indicated by solid lines, and matches to unselected variants indicated by dashed lines. The selected variant Det‐N‐Adj (which is disharmonic) has the same number of matches as the unselected variant Adj‐Det‐N (which is harmonic), and the tie is resolved randomly.

Fig. 5c shows one more example from the same iteration, now with harmony restored by the selection of Adj‐Det‐N. As the iteration continues, more and more multiword phrases with right edge N become available for sampling, gradually strengthening the preference for harmony even in more complex expressions.

**Fig. 5c:**
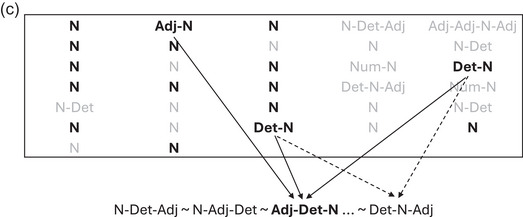
Linearization after 32 phrases of the Norwegian corpus. The harmonic variant Adj‐Det‐N now has decisively more matches than other variants, due to the growing pool of previous phrases with right‐edge N.

Through the incrementation of sampling procedures such as 5a–c, most iterations converge strongly on harmonic word orders. In each of the 100 iterations over each language corpus, we record the proportion of multiword phrases that are harmonic. Harmony is defined by calculating at which edge (left or right) the noun is predominantly positioned in the current iteration, then calculating what proportion of all multiword NPs in the iteration have the N at this edge.

**Fig. 6:**
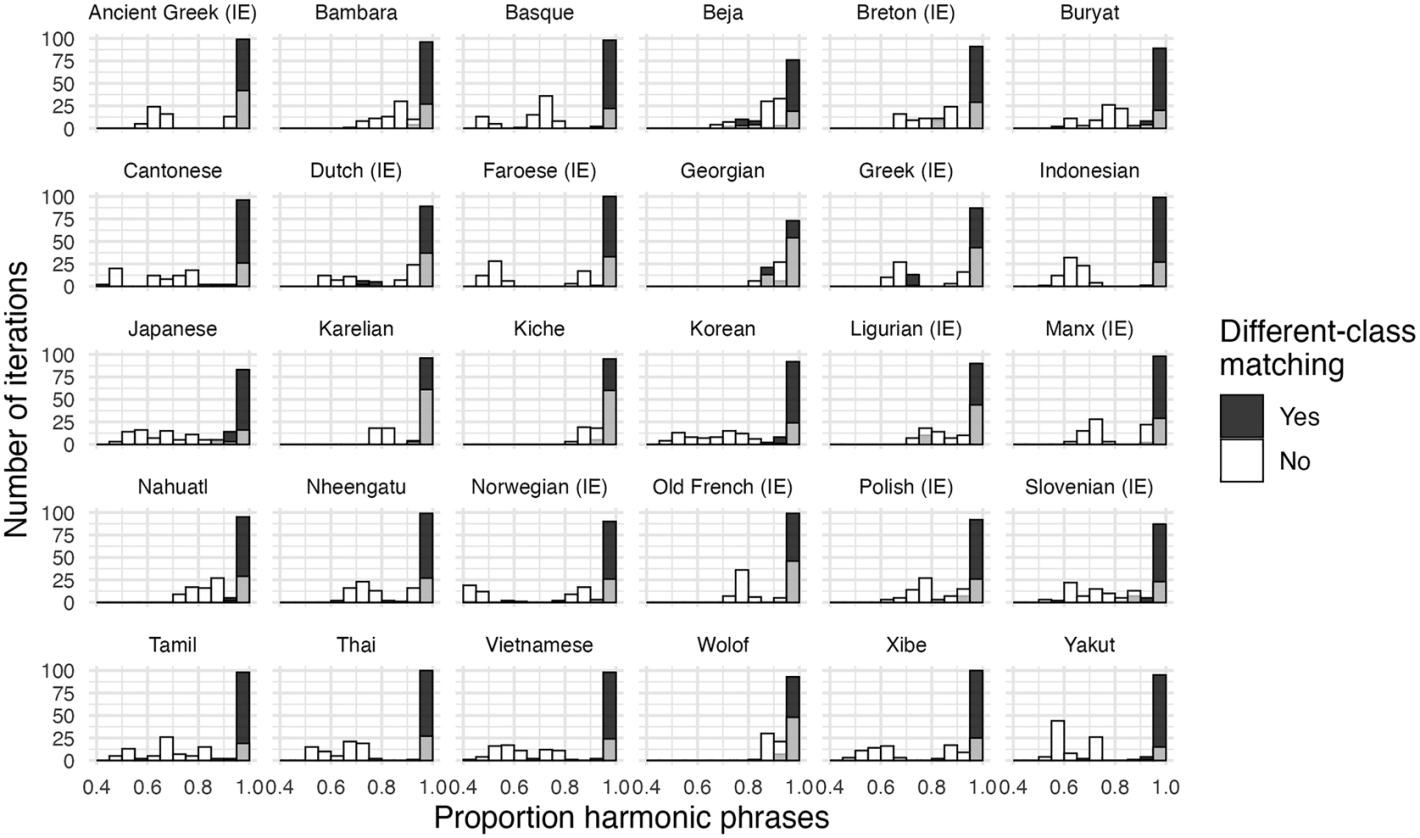
Histograms of harmony rates in the 30 illustrative corpora. The x‐axis indicates the proportion of harmonic phrases in an iteration, from 0.4 to 1.0, and the y‐axis indicates the numbers of iterations that fall into each proportion bin. Black bars illustrate the outputs of a replication process with different‐class matching, white bars illustrate the baseline comparison with only same‐class matching, and overlaps between the two display as gray coloring. In all corpora, phrasal replication with different‐class matching results in an overwhelming preference for harmonic order. By contrast, the model with only same‐class matching produces little or no preference for harmony.

Fig. 6 illustrates degrees of harmony for the 30 illustrative corpora. The figure also compares the main model of replication‐with‐modification against a baseline process, where words of the same class are matched to the same linear positions, but there is no matching between different word classes. This confirms the importance of different‐class matching, by illustrating how much harmony can be expected from purely same‐class matching of mostly short phrases. For most languages, almost all of the 100 iterations of the main model are clustered toward the right edge of the graph, indicating almost perfectly harmonic word orders. In the baseline process, without influence between word classes, we find a wide range of harmony rates between 0.4 and 1.

### 5.3 Harmony depends upon statistical properties of natural language

We have now seen that a simple replication algorithm, with mutual influence between words of different classes, produces harmonic word orders from natural corpus data. But it is also important to note that harmony depends on the statistical profile of these natural language samples. This shows that harmonic order is not a logical necessity of the replication algorithm, but a particular consequence of word‐class distributions in natural language.

**Fig. 7:**
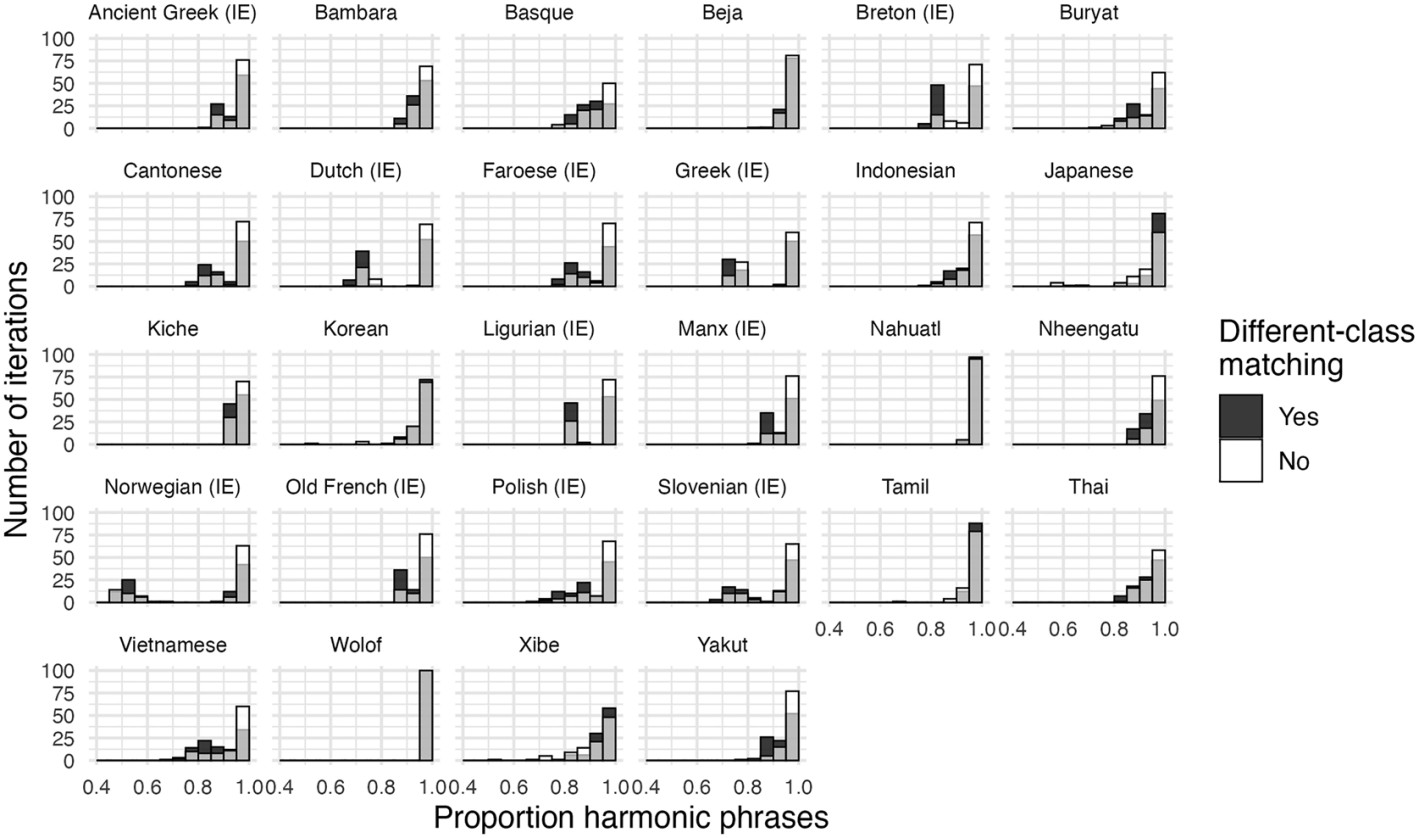
Histograms of determiner harmony in 28 corpora. Harmony preferences are weak or absent relative to determiners, confirming that the higher frequency of nouns plays a crucial role.

First, inspection of the less frequent word classes Det, Adj, and Num shows that none of them is consistently positioned at an edge. This means that word‐order harmony is only generated for the most frequent word class, N. This is illustrated for determiners in Fig. 7, while similar results for adjective and number are provided in the Supplementary Analyses. The figure shows degrees of “determiner harmony,” that is, proportion of determiners positioned at a consistent edge in multiword phrases, mirroring the measure used for nouns above. There is still a high degree of harmony for some languages, which reflects the fact that determiners are usually the second‐most frequent word class. But there is significantly less harmony than there was for nouns. Furthermore, there is no longer a clear contrast between the main model and the baseline process. Determiner harmony only emerges consistently in languages like Nahuatl and Wolof, where almost all NPs in the corpus data consist maximally of {N, Det} (see Fig. 3), which means that determiner harmony emerges almost inevitably from the statistical profile, and is equally generated by the baseline process. By contrast, determiner harmony is weaker in languages that have a high frequency of adjectives or numbers, relative to the frequency of determiners. This is the case in Greek and Vietnamese. Note that Georgian and Karelian are absent from this figure, as these UD corpora do not have any determiners in NPs.

Second, we can see that harmony depends upon the predominance of short phrases (see Fig. 4). If we run the model on an artificial corpus in which phrases of 1–4 words are all equally frequent, we no longer find a clear tendency toward harmony. We generated a corpus of 1000 expressions, using expression types {N}, {N, Adj}, {N, Adj, Num}, and {N, Adj, Num, Det}, where each of these expression types is equiprobable. In this artificial corpus, nouns are still the most frequent word class, but there is no tendency toward shorter phrases, in contrast to the distribution we observed for natural languages.

**Fig. 8:**
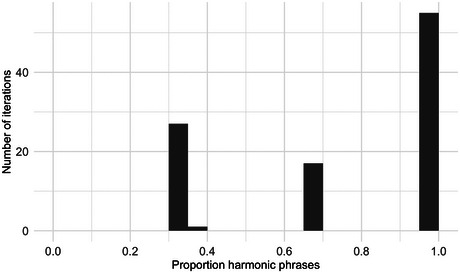
Histograms of harmony rates in 100 runs on an artificial corpus, where NPs have no tendency toward low complexity. The greater complexity of NPs in this artificial corpus destroys the preference for harmonic ordering.

Fig. 8 illustrates 100 iterations on the artificial corpus. We no longer find a strong tendency to harmony, but instead, iterations converge on one of three outcomes. One possible outcome is that all three of the less frequent classes (Det, Adj, Num) end up on the same side of the N, in a generalized schema such as Adj‐Num‐Det‐N. This generates harmonic outcomes, indicated by the cluster of iterations at the right edge of the graph. But there is now also a substantial number of iterations that diverge from harmony, with one non‐noun class occurring on the opposite side to the others. This proportion of disharmonic outcomes is expected to increase, as the complexity of phrases in the corpus increases.

### 5.4 Analytical proof of frequency‐based harmony

Thus far, we have seen evidence for frequency‐based harmony in computational implementations of the algorithm. But given that we claim this to be a fundamental principle, it is also desirable to prove analytically that the most frequent word class *must* tend toward an edge. To this end, we developed a formal mathematical description of harmony. This does indeed reveal fundamental harmonic biases in the phrasal replication algorithm, for example, finding that replication processes can shift from disharmony to harmony, but not vice‐versa. The formal description also allows some exact calculations of expected degrees of harmony. The full description is included in the data repository, while an outline is provided here.

**Fig. 9:**
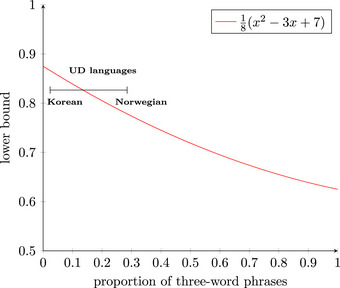
Lower bound for expected proportion of harmonic phrases, where phrase complexity varies between two words and three words. For all proportions of three‐word phrases, there is a general preference for harmony, but this decreases as three‐word phrases increase in frequency. This supports the proposed association between phrasal simplicity and harmony. Also shown is the range of frequencies for three‐word phrases in the natural language corpora, which lies between 0.024 (Korean) and 0.285 (Norwegian).

Frequency‐based harmony can be formalized as multiset unordered expressions, for example, {N, A, B} or {N, A, A}, which are linearized into phrases such as N‐A‐B, N‐B‐A, and so on. Replication‐with‐modification of previous phrases is formalized as a mapping function between phrases. We can then calculate probabilities of harmonic ordering in randomized sequences of phrase production, though combinatorial complexity makes such calculations impractical for systems with longer phrases or larger numbers of word classes. We, therefore, calculate probabilities for a language with three word classes (N and two other classes) and NPs consisting of no more than three words. As above, different‐class matching is allowed when same‐class matching has been exhausted. This allows us to compute a precise lower bound for the expected proportion of multiword phrases that are harmonic. This lower bound depends on the proportion of three‐word phrases compared to two‐word phrases, and can be calculated precisely using the quadratic formula illustrated in Fig. 9. This equation is derived from our formal mathematical description of phrasal replication, in the Supplementary Material. It shows that as the proportion of three‐word phrases increases, we get a lower degree of harmony. In the natural language corpora under consideration (see Fig. 4), the proportion of three‐word phrases among all multiword phrases ranges from *x* =  0.024 for Korean to *x* =  0.285 for Norwegian. Inserting these values into the functional equation shown, we obtain that the expected proportion of harmonic phrases is at least 0.867 for Korean and at least 0.790 for Norwegian.

In summary, for phrases of up to three words, given the range of three‐word frequencies in the corpus data, we can prove that they will tend toward harmonic ordering under our replication algorithm. While combinatoric complexity makes such calculations impractical for longer phrases or more word classes, this calculation already demonstrates that shorter phrases tend toward more harmony.

## 6 Relevance of the model to natural language processes

By reconceptualizing harmonic word order as the positioning of the most frequent word class at an edge, we have shown that harmony emerges spontaneously from a simple phrasal replication algorithm. The replication process is based on words of the same class appearing in the same relative positions, though crucially, a kind of approximate replication can also occur where words of different classes are positioned in the same way, as, for example, when a previous phrase X‐Y matches a new phrase X‐Z. The emergence of harmony is also driven by the characteristic statistical profile of NPs in the corpus data, where one word class is much more frequent than the others, and shorter phrases are more frequent than longer phrases. This draws a fundamental connection between harmonic order and the statistics of natural language.

The simplicity of this model makes it an attractive explanation for parallel harmonic order, and suggests that the notion of headedness, with a grammatical rule controlling head‐dependent ordering, is unnecessary for explaining word order. A model based on approximation and variation is also attractive because it produces harmony as a probabilistic tendency, which better fits the empirical data (Hawkins, 1980). This also opens the way for interaction with competing probabilistic forces, which might explain why not all phrases in all languages are harmonic.

In this section, we will show that frequency‐based harmony is not just parsimonious, but also plausible as a mechanism of natural language, by demonstrating some extended implementations, and discussing its compatibility with psycholinguistic models. We first address the applicability of the algorithm to other grammatical structures beyond the NP, then turn to questions of syntactic learning and production, with additional simulations that integrate fuzzy word classes and multiword chunking. Another area in which compatibility might be considered is in processes of iterated learning among generations of individuals, and we include a simulation of this in the Supplementary Analyses.

### 6.1 Harmony in other grammatical structures

Our initial demonstration focused on NPs, as the prime example of cross‐linguistically attested parallel harmony. However, frequency‐based harmony is not intrinsically linked to NP grammar, but instead is facilitated by statistical properties of which the NP provides just one example. Therefore, we should expect frequency‐based harmony to apply to other grammatical structures.

At a lower structural level, frequency‐based harmony could apply to morphological affix positioning. In complex word structures, we would expect the stem to be the most frequent morphological class, and affix classes less frequent. This would predict that stems tend to be positioned at the edge of word structure, with multiple affixes arranged mostly on one side of the stem. This might interact with other effects, such as a preference for suffixes over prefixes (Cutler, Hawkins, & Gilligan, 2009; Himmelmann, 2014; Martin & Culbertson, 2020). We do not know of any existing research that directly addresses this prediction, nor do we know of any multilingual corpora that might facilitate an implementation, but this could be a fruitful direction for further research.

**Fig. 10:**
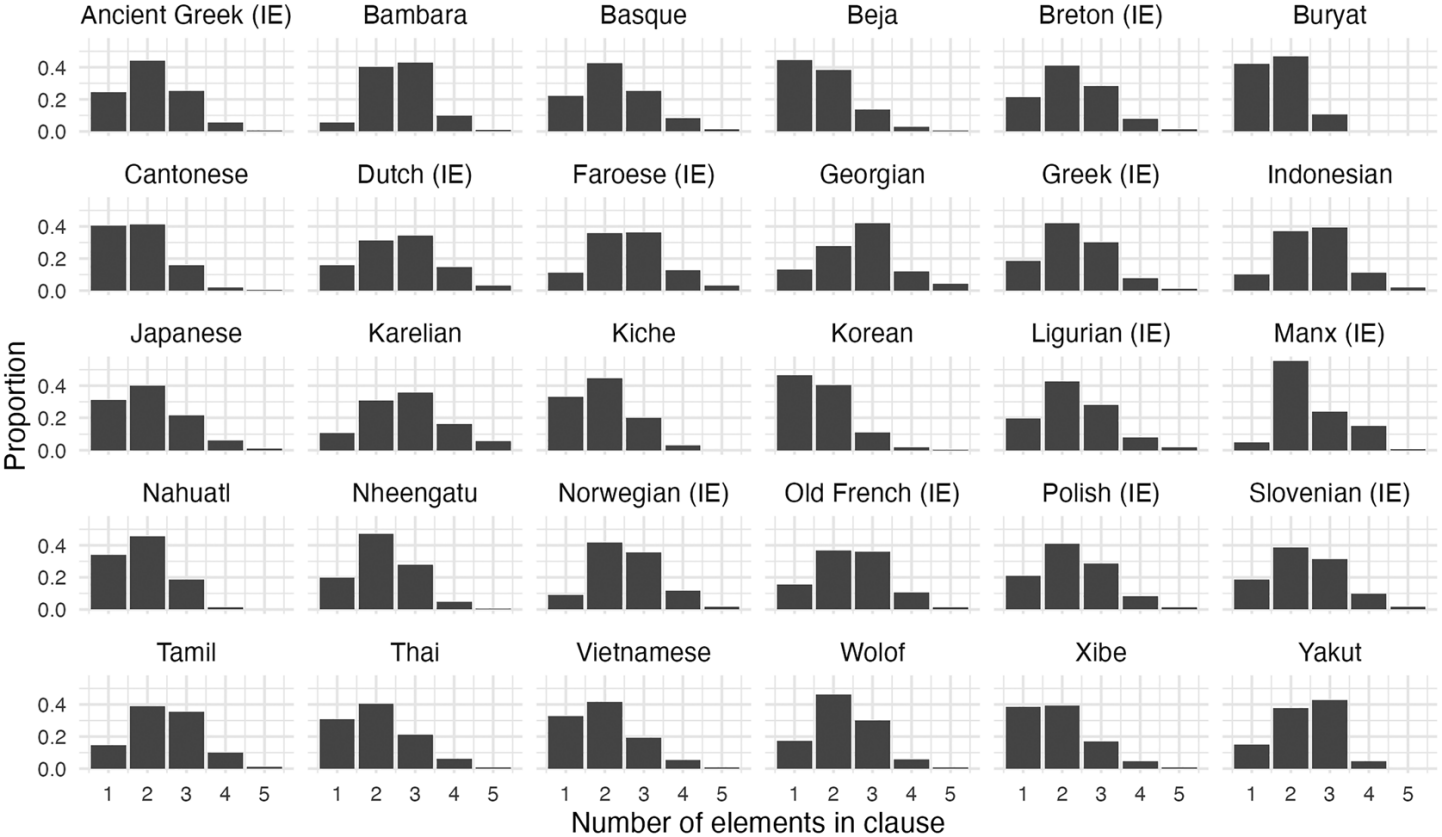
Clausal complexity for the 30 illustrative corpora, counting the verb and its arguments as constituents. Phrasal complexity is higher in clauses than in NPs, which should result in a lower degree of harmonic ordering.

At a higher structural level, clauses exhibit another type of parallel dependency. Subject, object, and other arguments are typically analyzed as multiple dependents of the same head, namely, the verb. Thus, a clause exhibits parallel harmony when multiple arguments occur on the same side of the verb, as in S‐O‐V or V‐S‐O (Dryer, 1997). Oblique arguments or adpositional phrases can also be analyzed as contributing to clause‐level harmony (Dryer, 2009). We can demonstrate frequency‐based harmony with these clausal elements much as we did for NPs above. First, we extract unordered clausal expressions from the UD corpora, this time targeting verbs and their associated subject, object, and oblique arguments. For example, the corpus sentence *The dog chased the cat* would yield an unordered set of symbols {O, S, V}, and the sentence *She placed it on the table* would yield {O, Obl, S, V}. All the expressions thus extracted contain exactly one V symbol, maximally one S symbol, maximally one O symbol, and any number of Obl symbols. Fig. 10 illustrates the complexity of these clausal structures in our sample languages. Complexity here is somewhat higher than what we found for NPs. Whereas NPs were usually between 1–2 words, with a substantial minority of 3‐word clauses but rarely longer than this, the clause elements we extract here usually number 1–3 elements per clause, with a substantial minority of 4–5 element clauses in many languages such as Dutch, Karelian, and Manx. Given that frequency‐based harmony depends on low average complexity of phrases, we should, therefore, expect to find a lower degree of harmony in clauses compared to NPs.

**Fig. 11:**
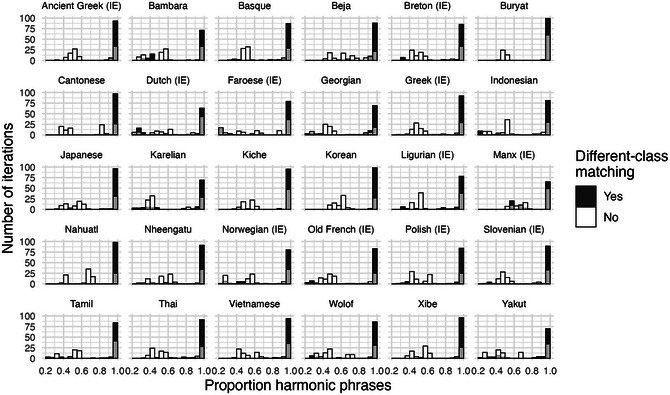
Histograms of clausal harmony rates in the 30 illustrative corpora. As with NPs, a replication process with different‐class matching again produces mostly harmonic orders, though the tendency is somewhat weaker here due to the higher average phrasal complexity. Note that the x‐axis limits here are different from the NP results in Fig. 6, as the clausal implementation produces more deeply disharmonic iterations.

Using the UD clausal expressions, we run exactly the same replication algorithm as we did for NPs. Fig. 11 shows the results, again comparing the main process that allows different‐category matching against a baseline with only same‐category matching. The majority of iterations with different‐category matching again produce harmony rates close to 100%. However, in the languages with more complex clause structures, such as Dutch, Karelian, and Manx, the proportion of strongly harmonic iterations is somewhat reduced. The greater disharmony in clause ordering follows our principle of phrasal complexity: since clausal expressions are on average more complex than NPs, the degree of harmony for clauses is somewhat lower than it was in the NPs.

The clausal implementation suggests that the frequency‐based explanation for harmony is not specific to NPs, but should be applicable to any other structure that meets the fundamental statistical properties outlined in Section 5.3. However, although our implementation might suggest a preference for parallel harmony on the clausal level, in natural languages, the frequency‐based harmony effect might here be overshadowed by a variety of other factors. Many different mechanisms have been proposed to influence clausal order (Song, 2018), including animacy or agency effects (Sauppe et al., 2023), communicative efficiency (Gibson et al., 2013), or pragmatic salience (Mithun, 1992). Furthermore, clause production in natural language is often considered to be incrementally planned (Chang et al., 2008), in contrast to the holistic ordering implemented in our model, which may be more applicable to NPs (Martin, Crowther, Knight, Tamborello, & Yang, 2010; Roeser, Torrance, & Baguley, 2019).

Despite the theoretical complexity of clausal ordering, we can still make a tentative prediction from frequency‐based harmony: in languages where S and O arguments are less frequently overt, they should have a stronger tendency to different‐category matching, and therefore, be more likely to appear on the same side of the verb. Thus, harmonic basic orders such as S‐O‐V and V‐S‐O should correlate with more argument omission. This prediction is compatible with some previous work arguing that harmonic S‐O‐V order is preferred in languages with less overt arguments, compared to those with more overt arguments, which prefer disharmonic S‐V‐O (Hahn & Xu, 2022; Luk, 2014; Ueno & Polinsky, 2009). Further research might aim to explicitly model replication‐with‐modification as part of the mechanism generating clausal ordering differences in languages with different frequency profiles.

### 6.2 Fuzzy word classes

In our initial implementation, we maximized simplicity by representing word classes as discrete symbols {N, Adj, Num, Det}, which, however, can be matched to other symbols in phrasal replication, if same‐class matching has been exhausted. Arguably, a more realistic model is one in which word classes are not discrete symbols, but instead are latent, fuzzy categories, with degrees of similarity between exemplars. As mentioned above, this is the approach taken in some recent typological investigations of word classes (Keizer, 2023). It is also more plausible from the perspective of language acquisition, where children must discover syntactic categories from linguistic input and perceptual experience. In this spirit, some computational studies model early learning based purely on individual words, without any syntactic category annotation (e.g., McCauley & Christiansen, 2019). There is also a great deal of cognitive modeling work on category formation from individual exemplars (e.g., Love, Medin, & Gureckis, 2004; Nosofsky, 2011). It is, therefore, of interest to test whether frequency‐based harmony can be demonstrated with latent, fuzzy categories. To do this, we created an alternative implementation that models words as points distributed in a “similarity space,” without any overt category labeling in the replication mechanism. Instead, there are latent syntactic categories {N, Adj, Num, Det} underlying the similarity distributions, allowing us to evaluate whether harmony emerges for the most frequent fuzzy category, N.

**Fig. 12:**
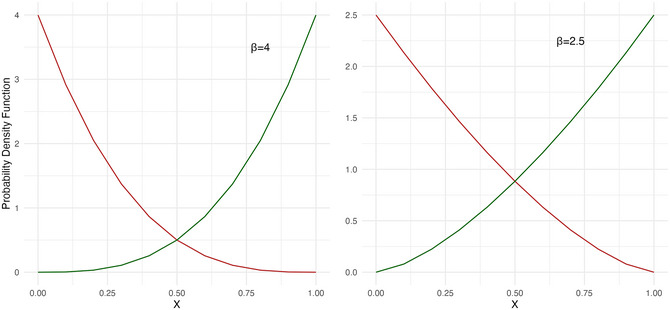
Beta distributions used to model word classes as clusters in multidimensional space. The x‐axis shows an example of one spatial dimension (say, nouniness), and the y‐axis shows the density of words in this dimension. In the nouniness dimension, nouns tend toward high values (green line) and all other classes tending toward low values (red line). The beta parameter can be used to produce tighter clustering and less overlap (left), or looser clustering and more overlap (right).

Modeling word order with a more complex representation of syntactic categories threatens to drastically increase the complexity of the model. We, therefore, design our fuzzy categories in the simplest way possible. We use a four‐dimensional similarity space, in which words are distributed as spatial coordinates. The words labeled N, Adj, Num, Det in the UD corpus data are probabilistically assigned spatial coordinates in such a way that they form gradient clusters in the four‐dimensional space, with word matching and replication now based on spatial proximity of individual words, as opposed to discrete word class labels. This is compatible with some recent experimental work exploring grammatical ordering based on similarity (Herce et al., 2023; Mansfield et al., 2022). We here remain agnostic as to what sorts of similarity underlie word classes (but see, e.g., Aarts, 2007; Gärdenfors, 2014), and model the similarity space using beta distributions to create clustering at the extremes of each of the four dimensions, such that each latent class has high values in one dimension, and low values in the other three dimensions. For example, nouns are spatial coordinates with high values in the “nouniness” dimension, while words of all other classes have low values in the nouniness dimension, and so on for each of four dimensions. This design introduces only one free parameter to the model, namely, a single beta parameter used for the spatial distributions, which determines the tightness of the clusters in the similarity space. Fig. 12 illustrates the effect of the beta parameter in a single dimension, for example, the nouniness dimension in which noun words have high values, and all non‐noun words have low values. The left panel illustrates β =  4, where there is only a small degree of word‐class overlap in the center of the graph. In the nouniness dimension, this would generate very few word pairs of Ns and non‐Ns with a similar nouniness value, and therefore, only a few similarity matches between Ns and non‐Ns. The right panel illustrates β =  2, generating a much greater degree of overlap between latent word classes, and therefore, more different‐class matching.

In our discrete‐classes model, phrase replication first matched words of the same class, and then matched any unmatched words to remaining words of different classes. In the fuzzy version, the algorithm instead matches words according to spatial proximity. Given words *w*_T1_ … *w*_Ti_ in the target expression, and words *w*_S1_ … *w*_Sj_ in a sampled previous phrase, matching begins with whichever pair *w*_T_, *w*_S_ has the shortest distance. This pair of words is then excluded from further matching. Matching proceeds in this way, according to the ranked proximity of pairs, until either the target expression or the sampled phrase has been entirely matched. All other aspects of the implementation remain as before.

The fuzzy‐classes implementation, like the discrete‐classes version, produces an overwhelming tendency toward harmonic orders. Note that harmony rates can still be calculated based on the latent (fuzzy) noun class, even though there are no overt syntactic labels used in the replication algorithm, just gradient spatial distributions. As shown in Fig. 13a, the tight similarity clusters with β =  4 produce close to 100% harmony in almost all iterations. However, if clustering is loosened to β =  2.5, as shown in Fig. 13b, the harmonic tendency is substantially attenuated. Looser classes gradually undermine the harmonic tendency, likely because they create less consistent ordering.

**Fig. 13a:**
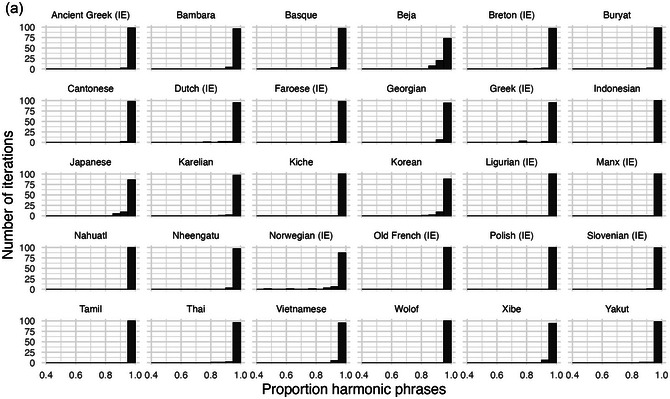
Histograms of harmony rates with fuzzy word classes and β =  4. The small amount of semantic overlap between word classes is sufficient to produce a strong harmonic tendency.

**Fig. 13b:**
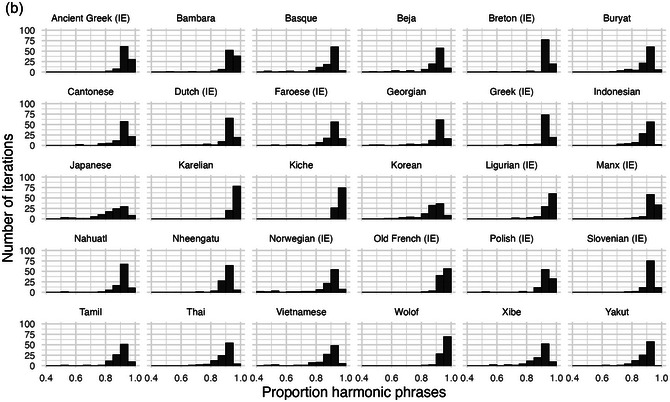
Histograms of harmony rates with fuzzy word classes and β =  2.5. When word classes have greater overlap, word‐class matching is weakened and so is the harmonic tendency.

The results of the fuzzy‐class implementation indicate that frequency‐based harmony does not depend on the specific implementation with same‐class matching followed by different‐class matching. Rather, frequency‐based harmony is expected to emerge in a range of models, including those with gradient word classes. The only necessary conditions are that there are *some* kind of word classes (without which the definition of harmony becomes meaningless), and that word‐matching in replication should favor same‐class matching, while also allowing some different‐class matching.

### 6.3 Locality and chunking of multiword sequences

As mentioned above, parallel harmony is in competition with locality effects, which intrinsically tend to violate parallel harmony. With multiple parallel dependents, locality favors sequences such as X‐N‐Y, where dependents are maximally close to the head by being arranged symmetrically on both sides. This directly competes with the harmonic principle found in structures such as N‐X‐Y, and thus offers a potential explanation for why natural languages exhibit only partial harmony (Jing et al., 2022). The clash with locality makes our need for a convincing theoretical explanation of parallel harmony all the more urgent. But we should, therefore, also seek an explanation of harmony that can be integrated with locality effects. In particular, we should expect that integrating the two principles will reduce the degree of harmony produced.

Locality has been proposed in two different versions, an earlier one claiming that it is driven by dependency relations, and a more recent one claiming that proximate linearization of words is driven by high mutual information (MI) between words (Futrell, 2019). In the domain of the NP, evidence for this “information locality” has been adduced by showing that the classes Adj, Num, and Dem tend to have different degrees of MI with nouns, in the ranking Adj > Num > Dem, and this matches typological tendencies for linear proximity to the noun (Culbertson, Schouwstra, & Kirby, 2020; see also Hahn, Degen, Goodman, Jurafsky, & Futrell, 2018). Thus, adjectives tend to be at least as close to the noun as number words, which tend to be at least as close to the noun as demonstratives (Dryer, 2018). We here implement this informational version of locality, incorporating it into our NP linearization algorithm.

One way of modeling information locality is via chunking, where words with high MI are output as contiguous multiword chunks (Mansfield & Kemp, 2025; McCauley & Christiansen, 2019). This produces a correspondence between MI and linear proximity (for alternative models of information locality, see Futrell, Gibson, & Levy, 2020; Hahn, Degen, & Futrell, 2021). Chunking has been proposed as a psycholinguistic solution to the problem of parsing rapid sequences of words in real time (Christiansen & Chater, 2015), though it could also aid syntactic production by more efficiently retrieving frequent multiword chunks (Mansfield & Kemp, in press). Chunking is also at the heart of one psycholinguistic model, the Chunk Based Learner (McCauley & Christiansen, 2019), which demonstrates that sentences ordered based on chunk‐learning match the sentences spoken by children to a significant degree.^6^

To implement a chunky version of the replication algorithm, we simply add a filter on the selection of variant word orders. We treat pairs of words with high MI as inseparable chunks, thus excluding any variant in which such a pair would be nonadjacent. For computational tractability, we implement only bigram chunks. Obtaining accurate and comprehensive MI measures for word pairs is a significant challenge (Culbertson et al., 2020), and for this, we used massive corpus data available from the Google Books project (Goldberg & Orwant, 2013; see Supplementary Analyses for details). Given the computational demands of the MI calculations, we limit this analysis to English only. We thus use the English UD corpora as our NP source data, in the same way as above, but now add an additional step in the processing of each NP, checking MI values for all word pairs and assigning chunks accordingly. This introduces one free parameter into the model, namely, the MI threshold at which a word pair is treated as a chunk. Lower MI thresholds generate more chunking, and thus stricter locality conditions, which should in turn create more harmony violations.

**Fig. 14:**
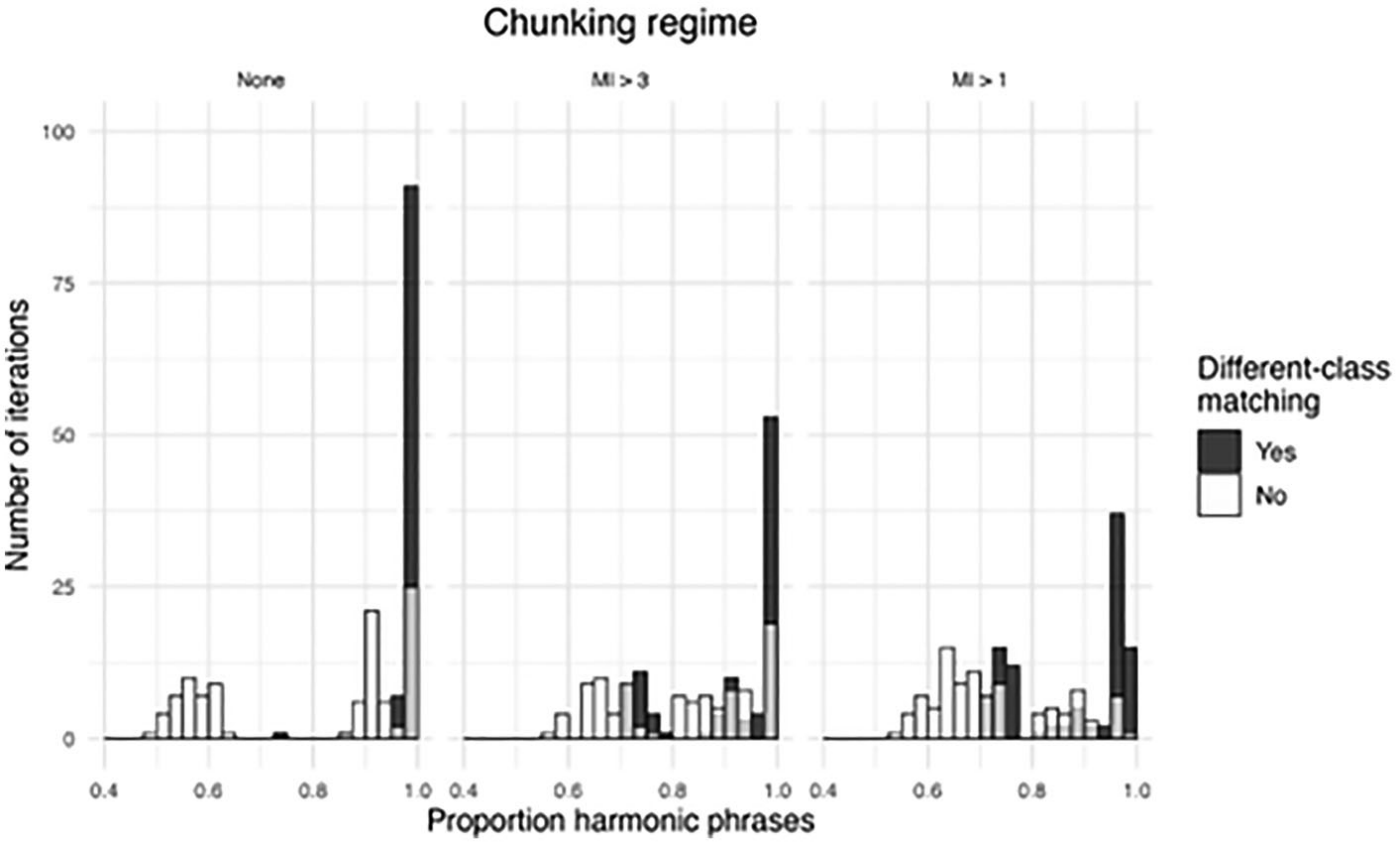
Histograms of harmony rates in English with chunking at MI > 3 and MI > 1. A moderate amount of chunking (at MI > 3) slightly weakens harmony, while still maintaining an overall harmonic tendency. More frequent chunking (at MI > 1) progressively undermines the harmonic tendency.

The chunky version of the replication algorithm works as expected. The left panel in Fig. 14 shows the degree of harmony produced by our main (nonchunked) algorithm when run on English corpus data. Like the other languages, this produces almost perfect harmony in most iterations. The middle panel shows the results with a small amount of chunking, treating word pairs with MI > 3 as chunks. As expected, this somewhat reduces the degree of harmony, since it occasionally favors X‐N‐Y structures over N‐X‐Y structures. The right panel shows a lower threshold of MI > 1, which provokes more chunking and thus produces more disharmonic iterations. These results capture the trade‐off between harmony and locality, and suggest that locality may provide one explanation for why harmony is not consistently exhibited in natural languages.

### 6.4 Compatibility with psycholinguistic experiments and models

We turn now to the compatibility of frequency‐based harmony with behavioral experiments in artificial language learning, and general models of syntactic learning and production. We argue that, although our replication algorithm does not represent the complexities of psychological phenomena, it is nonetheless compatible with such phenomena. This makes our proposal not just parsimonious, but also plausible as an explanation for harmonic ordering in natural language.

Behavioral experiments on harmonic order have yielded results that are compatible with frequency‐based harmony. A series of such experiments have been conducted using an artificial language learning paradigm, finding that participants prefer harmonic ordering when learning novel NP‐like structures, even if their native language has disharmonic NPs (Culbertson & Newport, 2015; Culbertson & Newport, 2017; Culbertson et al., 2012; Culbertson et al., 2020). Participants are presented with images and labels in a miniature invented language, with labels consisting of either {N, Adj} or {N, Num} word pairs. The training phase uses variable word orders, and the test phase investigates whether participants’ own productions in the artificial language exhibit a shift toward harmonic ordering, in comparison to their training data. The experiments found a bias toward harmony in adult English speakers (Culbertson et al., 2012), English‐learning children aged 6–7 (Culbertson & Newport, 2015), and adult speakers of French and Hebrew (Culbertson et al., 2020).

Frequency‐based harmony provides a neat explanation for the artificial language learning results. Every training exposure includes an N label, while only half the exposures include an Adj, and half include a Num. Therefore, Ns are the most frequent class (as in natural language corpora), and participants encounter scenarios where, for example, they have previously learnt a phrase of the form N‐Adj, and they are then presented with a phrase containing the elements {N, Num}. A natural solution for participants, when they “match” the new phrase with the old one, is to match N with N, expecting these to be in the same linear position, then match Adj with Num, despite their different word classes. A learning mechanism of this type would then learn N‐Num more efficiently than Num‐N, and subsequently tend to produce harmonic phrases such N‐Adj and N‐Num, as reflected in the experimental results. One way of summarizing the experiment is that participants readily treated novel nouns as “the same” for the purposes of linear ordering, but furthermore treated adjective and number words as “the same” when shifting between phrase types. This is captured by different‐class matching in our basic phrasal replication algorithm. The relevance of our model to novel sequence learning is further reinforced by a recent follow‐up in which participants exhibited harmonic ordering of Adj‐N and V‐N phrases, but only when there was semantic similarity between the Adj and V words in question (Wang, Kirby, & Culbertson, 2023). This is captured by similarity‐matching between fuzzy categories, in the gradient version of our algorithm.

We can also consider psycholinguistic relevance from the point of view of general computational models of syntactic learning and production. Our phrasal replication principles appear to be compatible with a wide range of usage‐based psycholinguistic models, including ANNs, which sample from one set of sentences, then must replicate similar ordering on a set of test sentences (e.g., Chang et al., 2006; Chang et al., 2008; Everbroeck, 2003; Lupyan & Christiansen, 2002). Such models are “usage‐based” because they produce new sentences by replicating the structure of input examples, implementing probabilistic selection over variant orders, and using some variety of approximation or extrapolation to enable the production of new expressions beyond the input. Each of these design features is mirrored in our algorithm, which at the same time minimizes its commitments to any particular training regime, probabilistic selection process or evaluation target.

## 7 Conclusion

Our simple phrasal replication algorithm offers a parsimonious explanation for parallel harmonic word order, which is one of the most widely discussed and demonstrated tendencies of natural language syntax. We propose that harmonic ordering is really a frequency effect: as replication converges on a phrase structure with a consistent linear order, the most frequent word class tends to be at one edge. This is because lower frequency classes are more subject to different‐class matching, which tends to position them on the same side of the most frequent class. We first demonstrated such a process in the simplest possible form, then showed how it can be integrated with features of language processing such as fuzzy word classes and multiword chunking. Since the algorithm can be easily integrated with these processes, and no doubt many others, we argue that it is not just parsimonious, but also psychologically plausible as an explanation for parallel harmonic order.

The most unorthodox assumption we have made is that word classes are not fully discrete. Instead, we assume that words of different classes may influence each other to some extent, for example, via gradient similarity measures that apply both within classes and between classes. We hope that the success of this approach in explaining harmonic order will promote further research on how syntactic structure can be modeled in terms of similarity relations between words. If a similarity‐based syntax has the power to explain harmonic word order as a consequence of basic statistical patterns, this is one reason, alongside existing typological evidence, to move away from word classes as fully discrete categories.

## Author contributions

JM conceived the study, developed the computational implementations, and wrote the manuscript. LSK added formal mathematical proofs and edited the manuscript.

## Notes

1
https://osf.io/m94en/

2 Other theories propose that all branching has the same underlying direction, though this is not directly observable due to intervening movement operations (e.g., Kayne, 1994). But movement operations add their own complexity to the grammar, and it is, therefore, debatable whether the underlying‐simplicity model really results in an overall simpler grammar or not (Culicover & Jackendoff, 2005: 47, 83).

3 This exploratory work (by the first author) has been archived in a research repository: https://osf.io/nq4hf/

4 The proposal does not account for situations where two or more word classes are obligatory in a phrase, or have equal highest frequency. We also assume that, in the majority of instances, each class appears maximally once per phrase. Multiple adjectives or determiners in a phrase are possible, but relatively rare.

5
https://osf.io/m94en/

6 The Chunk Based Learner uses transitional predictability, rather than MI, though the two are mathematically related through the concept of conditional probability.
